# Supplementary material for: Analysis of the Roles of the ISLR2 Gene in Regulating the Toxicity of Zearalenone Exposure in Porcine Intestinal Epithelial Cells
Source: Toxins (Basel). 2022 Sep 16;14(9):639. doi: 10.3390/toxins14090639 (PMC9506288; doi:10.3390/toxins14090639)
Supplement: Supplementary file 1 [file toxins-14-00639-s001.zip › toxins-1879834-supplementary.pdf]

toxins

MDPI

Article

Analysis of the Roles of the *ISLR2* Gene in Regulating the Toxicity of Zearalenone Exposure in Porcine Intestinal Epithelial Cells

Zhenbin Bi, Xuezhu Gu, Yeyi Xiao, Yajing Zhou, Wenbin Bao, Shenglong Wu and Haifei Wang

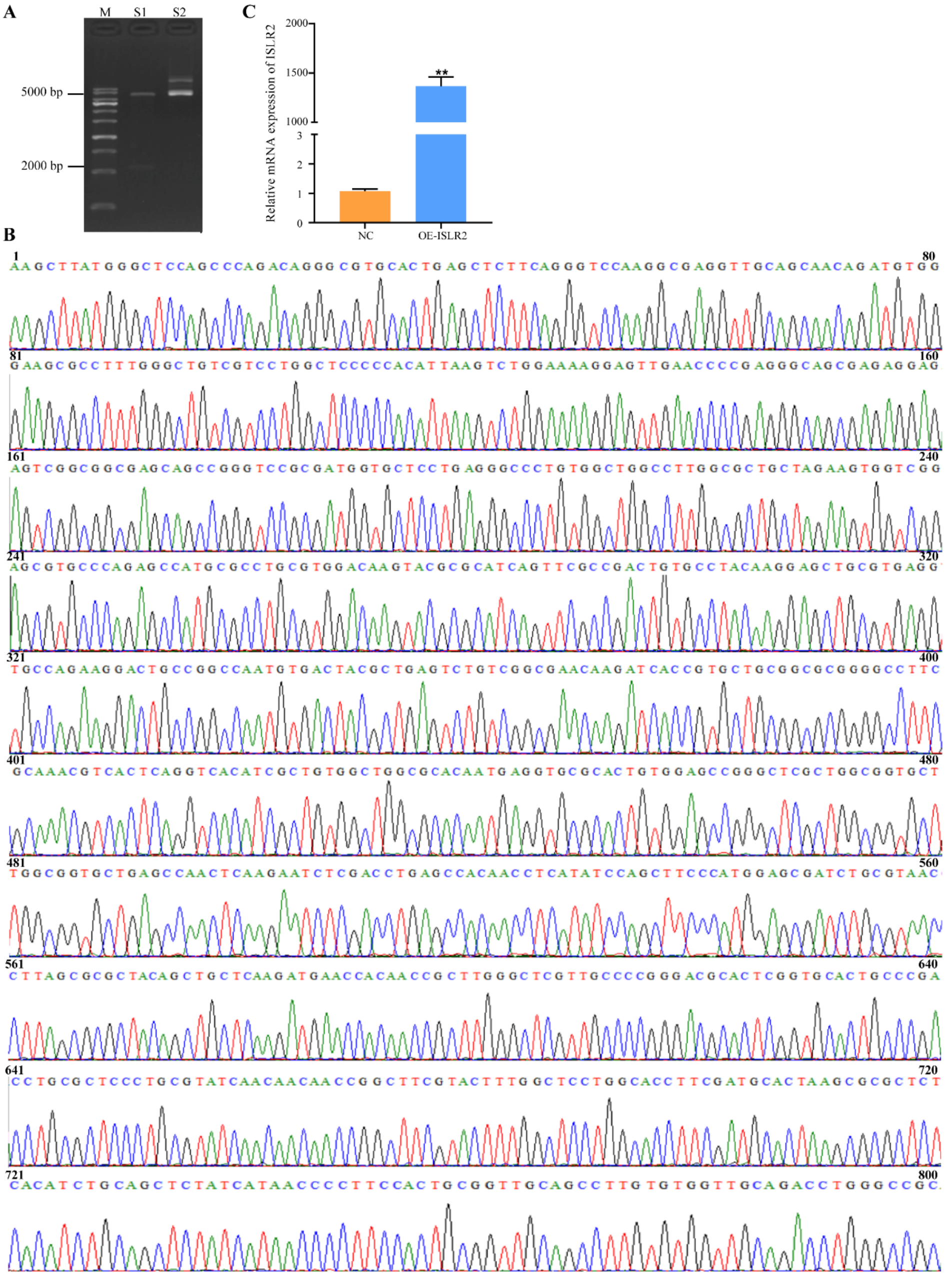

Figure S1. Construction of *ISLR2* overexpression cells. (**A**) Enzyme digested products of the plasmids overexpressing *ISLR2* by agarose gel. M: 1 kb ladder; S1: enzyme digested products of the plasmids with XhoI; S2: products of undigested sample; (**B**) PCR sequencing of the plasmids overexpressing *ISLR2*. (**C**) Efficiency of *ISLR2* overexpression by qRT-PCR.NC, cells transfected with an empty vector; OE-*ISLR2*, cells transfected with an *ISLR2* overexpression vector. Data were presented as mean ± SD. Significance compared with control, $^{**}P < 0.01$.
